# Fully-Gated Denoising Auto-Encoder for Artifact Reduction in ECG Signals

**DOI:** 10.3390/s25030801

**Published:** 2025-01-29

**Authors:** Ahmed Shaheen, Liang Ye, Chrishni Karunaratne, Tapio Seppänen

**Affiliations:** 1 Center for Machine Vision and Signal Analysis (CMVS), University of Oulu, FI-90014 Oulu, Finland; chrishni.karunaratne@oulu.fi (C.K.); tapio.seppanen@oulu.fi (T.S.); 2 Department of Information and Communication Engineering, Harbin Institute of Technology, Harbin 150001, China; yeliang@hit.edu.cn

**Keywords:** electrocardiogram (ECG), denoising autoencoder (DAE), convolutional neural network (CNN), self-ONN, U-Net, gated convolution, gated residual, attention, motion artifacts

## Abstract

Cardiovascular diseases (CVDs) are the primary cause of death worldwide. For accurate diagnosis of CVDs, robust and efficient ECG denoising is particularly critical in ambulatory cases where various artifacts can degrade the quality of the ECG signal. None of the present denoising methods preserve the morphology of ECG signals adequately for all noise types, especially at high noise levels. This study proposes a novel Fully-Gated Denoising Autoencoder (FGDAE) to significantly reduce the effects of different artifacts on ECG signals. The proposed FGDAE utilizes gating mechanisms in all its layers, including skip connections, and employs Self-organized Operational Neural Network (self-ONN) neurons in its encoder. Furthermore, a multi-component loss function is proposed to learn efficient latent representations of ECG signals and provide reliable denoising with maximal morphological preservation. The proposed model is trained and benchmarked on the QT Database (QTDB), degraded by adding randomly mixed artifacts collected from the MIT-BIH Noise Stress Test Database (NSTDB). The FGDAE showed the best performance on all seven error metrics used in our work in different noise intensities and artifact combinations compared with state-of-the-art algorithms. Moreover, FGDAE provides reliable denoising in extreme conditions and for varied noise compositions. The significantly reduced model size, 61% to 73% reduction, compared with the state-of-the-art algorithm, and the inference speed of the FGDAE model provide evident benefits in various practical applications. While our model performs best compared with other models tested in this study, more improvements are needed for optimal morphological preservation, especially in the presence of electrode motion artifacts.

## 1. Introduction

Cardiovascular diseases (CVDs) remain by far the leading causes of mortality, causing the death of an estimated 17.9 million lives worldwide in 2019 [1]. The percentages continuously increase so that diseases such as ischemic heart disease and stroke were solely responsible for 9.1 million and around 7 million of the world’s total deaths in 2021, respectively [2]. Electrocardiogram (ECG) [3] is a well-known non-invasive technology that can be used in the diagnosis [4,5] and screening [6] of several cardiovascular diseases. The ECG signal is the measured electrical activity of the heart acquired by adhesive electrodes attached to the skin. However, ECG signals can be contaminated by several types of noises, such as baseline wander (BW), power-line interference (PLI), muscle artifacts (MA), and electrode motion artifacts (EM) [7]. If the signal is highly corrupted by these artifacts, especially during Ambulatory Electrocardiography [8] or Exercise Stress Test [5], the reliability of using ECG in diagnosis and screening drops significantly. Generally, motion artifacts are known to be the most difficult noise type to be eliminated from ECG signals [9]. Motion artifacts’ spectrum highly overlaps with the spectrum of ECG signals, and the morphology of motion artifacts resembles the morphology of ECG waves.

Several decomposition and filtering methods in the literature were utilized to reduce noise and motion artifacts or to separate the different components of the signal. Simple filters such as the traditional finite impulse response (FIR) [10] and the infinite impulse response (IIR) [11] filters are not sufficient for removing severe motion artifacts in signals like ECG since they introduce distortions in amplitude, duration, and morphology [7]. Other more complex filters, such as least mean squares (LMS) and recursive least squares (RLS), have been popular in filtering ECG signals [12,13,14], as well as other versions such as Kalman filters, Wiener filters, adaptive median filters, etc. [15,16,17,18]. However, most adaptive filters might perform sub-optimally in extracting signals with similar statistical features if they fully overlap in time [19]. Moreover, the used noise reference could be uncorrelated with the motion artifacts contaminating the signals [9]. Blind Source Separation (BSS) approaches, such as Principal Component Analysis (PCA) and Independent Component Analysis (ICA), can cause distortion to the extracted signals due to bad handling of the background noise and not using the a priori information of the signal [19]. Other algorithms, such as Empirical Mode Decomposition (EMD), can cause distortion at the beginning and the end of the QRS complexes in ECG signals, resulting in a wider QRS complex, which makes it inconvenient for the decomposition of such signals [7]. EMD had several issues, such as mode mixing, where the frequency between the decomposed intrinsic mode functions (IMFs) overlaps [20]. Moreover, it is difficult to identify which IMF represents the motion artifact and is required to be removed [9]. Other versions of EMD, such as ensembled EMD (EEMD) and complete EEMD with additive noise (CEEMDAN) [21], tackle these issues, yet the computational speed decreases as the number of ensemble trials increases [22]. Variational Mode Decomposition (VMD) [23] is another algorithm that can be used in ECG denoising and can overcome the mode mixing problem in EMD, yet optimal IMF selection and VMD parameters setting are difficult to obtain [24]. Although wavelet transform methods such as discrete wavelet transform (DWT) [25] and empirical wavelet transform (EWT) [26] are quite popular for denoising tasks, they can be insufficient for removing motion artifacts from contaminated ECG signals due to spectrum overlap [9].

The drawbacks of different filtering and decomposition approaches make the choice of one algorithm that is robust, accurate, and efficient at the same time challenging. Using deep learning (DL) algorithms has become popular for denoising tasks in bio-signals and usually outperforms the traditional algorithms. Many deep learning algorithms exist and have been used for ECG denoising. For instance, a four-layer deep recurrent neural network (DRNN) has been used in [27] to denoise ECG signals. Romero et al. [28] proposed a multibranch deep convolutional neural network (CNN)-based model (namely DeepFilter) inspired by the famous inception model [29] to remove baseline wander artifacts from ECG signals. Wang et al. [24] proposed a generative adversarial networks (GANs) model utilizing convolutional autoencoders for ECG denoising and restoration. Diffusion models were also proposed to be used in ECG signal denoising as in [30,31]. Diffusion models have been the state of the art for image generation, and they show great performance, yet they have the major drawback of very slow inference time and are usually very resource intensive. As denoising autoencoders (DAEs) [32] become more popular in ECG denoising, many works showed a major improvement compared with other deep learning models. For example, a fully convolutional denoising autoencoder (FCN-DAE) [33] was proposed by Chiang et al., which showed superior ECG denoising performance compared with deep fully connected and convolutional models. An attention-based convolutional denoising autoencoder (ACDAE) model proposed by Singh et al. [34] utilized efficient channel attention (ECA) [35] in the decoder to improve the denoising performance of the model. Chorney et al. [36] proposed a convolutional block attention module (CBAM) [37]-based denoising autoencoder model (CBAM-DAE) with a similar structure to the ACDAE model, yet the CBAM attention module was used in both the encoder and decoder. Following these models, other models that use a similar idea of using attention in denoising autoencoders emerged, such as the transformer-based convolutional denoising autoencoder (TCDAE) model proposed by Chen et al. [38]. In that model, gated convolutions and a block of transformer encoder were used in the encoder part of the model to provide state-of-the-art performance on the benchmark proposed by Romero et al. [28]. Very similar structures with minimal differences where transformer blocks are utilized in a denoising autoencoder for motion artifact removal in ECG signals were proposed in [39,40,41].

Although the most recent deep learning approaches seem to have outstanding performance and better generalizability compared with traditional algorithms, the state-of-the-art approaches are large, slow, and resource-intensive. Further, cleaner signals, especially in severe motion artifact cases, are needed for a more reliable diagnosis in athletes and ambulatory cases, which the available approaches still lack. Therefore, in this work, we propose a novel compact architecture that surpasses the state-of-the-art performance with lower computational cost. The proposed Fully-Gated DAE (FGDAE) model embraces the idea of gating and attention and also utilizes the use of non-linearity in feature extraction to overcome the severe noise artifacts in ECG signals.

## 2. Related Work

### 2.1. CNN and Self-ONN for Feature Extraction

CNNs have become the de facto standard for many tasks in deep learning [42]. CNN has the ability to perform efficient feature extraction rather than hand-crafted features while considering the locality of the features. This made CNNs especially suitable for many image and signal processing tasks. For instance, Kong et al. [43] proposed a polydirectional-CNN-based structure to extract spectral–spatial features for multispectral image compression. The method was tested on the Landsat-8 and the WorldView-3 satellite datasets, and three performance indicators, namely spatial information recovery, spectral information recovery, and visual effect, were evaluated. Experimental results showed that the proposed structure had strong robustness as the bit rate dropped off. Elhassan et al. [44] used a hybrid CNN-based feature extraction and fusion method to extract features from white blood cells for computer-aided acute myeloid leukemia (AML) diagnosis. A total of 15 types of white blood cells, including both normal and AML cancer cells, were involved, and an overall classification accuracy of 96.41% was achieved. Chaudhri et al. [45] proposed a multidimensional, multiconvolution-based feature extraction approach for a drift-tolerant, robust classifier for gases and odors. Experimental results on a public gas dataset proved the effectiveness of the proposed approach.

While deep learning models are usually preferred in many tasks, compact CNN models seem to be sufficient in many signal processing tasks. According to [46], compact 1D CNNs are better than deep 2D CNNs and deep 1D CNNs in terms of data requirement (i.e., less training data needed) and computational complexity. Compact 1D CNNs have especially been state-of-the-art in several biosignal processing applications, such as early arrhythmia detection in ECG, patient-specific ECG classification, and anomaly detection [46,47].

Self-Organized Operational Neural Networks (self-ONNs) are generative non-linear CNN alternatives [48]. They can approximate any arbitrary nodal function ψ for each kernel element using the *Q*th order truncated approximation (a.k.a., MacLaurin polynomial approximation) as shown in Equation (1),

(1) $$\psi \;{\left(x\right)}^{\left(q\right)}=\;\sum_{n\;=\;0}^{q}\frac{\psi^{\left(n\right)}\left(0\right)}{n!}x^{n},$$

where the coefficients $\frac{\psi^{\left(n\right)}\left(0\right)}{n!}$ are learnable parameters. When the parameter q=1, the self-ONN is equivalent to ordinary CNN. More technical and mathematical details can be found in [48,49]. Self-ONNs have been used for feature extraction, denoising, and other signal processing tasks, as well as CNNs, and showed extraordinary performance. For example, Malik et al. [50] compared the AWGN (Additive White Gaussian Noise) image denoising performances of self-ONN with those of CNN and BM3D (Block-Matching and 3D filtering). On average, self-ONN outperformed CNN and BM3D in terms of PSNR (Peak Signal-to-Noise Ratio). When the standard deviation of the noise increased, the gaps between self-ONN and the other two widened even more. Yamanouchi et al. [51] combined self-ONN and Cycle-GAN (Cycle-consistent Generative Adversarial Network) for X-ray image denoising. The self-ONN in this structure was used for feature extraction. Via an evaluation on 300 datasets with sesame salt noise, the proposed structure showed outstanding performances in terms of both PSNR and SSIM (Structural Similarity). Devecioglu, et al. [52] proposed an improved active fire detection method using an operational U-net, in which the authors replaced the convolutional layers with operational layers from self-ONN for feature extraction. The Landsat-8 dataset was used for evaluation, and experimental results showed that the proposed operational U-net outperformed the convolutional U-net significantly.

Self-ONNs were also used in ECG signal analysis. For instance, compact self-ONN models were used in [49,53] for patient-specific and inter-patient ECG classification, respectively, and showed outstanding performance compared with the state-of-the-art with a significant margin. Furthermore, Qin et al. [54] proposed a general pre-trained classification model based on self-ONN, where it showed outstanding performance compared with CNN architectures on the PTB-XL database for an inter-patient 71-class classification task. Also, an algorithm based on 1D self-ONN was proposed in [55] for peak detection in Holter ECG, and it was shown that the proposed model could significantly surpass the state-of-the-art deep CNN models with less computational complexity. These works clearly show the value of using self-ONNs instead of CNNs in denoising and feature extraction models.

### 2.2. Denoising AE and U-Net for ECG Denoising

Autoencoders (AEs) are powerful deep learning structures that can be used in several applications, such as dimensionality reduction, where they seem to outperform PCA and can generate more effective features. They can be used in denoising tasks in both images and signals. In fact, several types of autoencoders exist, and each type tries to address certain problems as discussed in [56]. Especially, AEs that utilize CNNs in their hidden layers can be suitable for extracting morphological features in signals. Denoising autoencoder (DAE) [32] is a type of AEs that aims to restore a clean version of a corrupted output. This type of autoencoder has been used in ECG denoising as in the FCN-DAE model [33]. A special structure of autoencoders that has been recently used heavily in ECG denoising tasks is the U-Net architecture [57]. The U-Net was initially proposed for biomedical image segmentation. Later, Jansson et al. [58] proposed using this architecture in source separation tasks, which opened the door for U-Net to be used in denoising tasks as well. Recently, the U-Net has been used as the core structure of DAE models such as the ACDAE model [34], CBAM-DAE model [36], TCDAE model [38], and others used in ECG denoising. Hence, it can be said that the U-Net is the core architecture of the state-of-the-art ECG denoising algorithms. The efficient progressive compression of the input signal features in the encoder and progressive decompression in the decoder help the model learn the key features of the signal while ignoring redundant features. Further, the use of the residual connection between the mirrored layers of the encoder and decoder helps retain the information flow and preserve the important features in the input signal that could be lost in the compression/decompression stages of the model.

### 2.3. Gating and Gated Convolutions

Gated Convolutional Networks (GCNs) [59] introduce a gating mechanism that can control the flow of information through the network. This mechanism is inspired by the gating units in recurrent neural networks (RNNs), such as Long Short-Term Memory (LSTM) units and Gated Recurrent Units (GRUs) [60], which regulate the information passing through the units based on the context. In GCNs, a gating mechanism is applied to the output of the convolutional layer. That is, the convolution operation is followed by a gating operation. The gating mask is generated using another independent convolution operation followed by an activation (i.e., typically a sigmoid function). The gating is done by element-wise multiplication between the gating mask and the output of the main convolutional layer. Therefore, it can be understood that the values in the gating mask control which features are allowed to pass through and which to suppress, dynamically adjusting the flow of information based on the input. In a gated convolutional layer with input *x*, the output *y* is computed as follows:

(2) $$y=Conv_{i}\left(x\right)⊙\sigma \left(Conv_{g}\left(x\right)\right),$$

where σ(·) is the sigmoid function, ⊙ denotes element-wise multiplication, and $Conv_{i}\left(\cdot \right)$ and $Conv_{g}\left(\cdot \right)$ are the convolution operations for input and gate, respectively. Gated convolutions can potentially improve the performance of denoising models by dynamically controlling the flow of information and emphasizing important features. In the ECG denoising task, gated convolutions can be particularly useful in distinguishing between the features of ECG signals and motion artifacts since they can dynamically adapt to different types of artifacts and suppress their related features. Gated convolutions were used in the model proposed by Chen et al. [38] along with other modules to denoise ECG signals, and state-of-the-art performance was reported. Hence, we try to utilize the idea of gated convolutions in our work as discussed later on.

## 3. Materials and Methods

### 3.1. Model Architecture

The different artifacts corrupting the ECG signal can be represented by mainly two components: additive and multiplicative components, respectively. For example, respiration motion causes the heart to change orientation and move from its original position (w.r.t. the measuring electrodes), causing amplitude modulation to the measured ECG [61]. Furthermore, the change in conductivity of the skin tissues due to chest movement and blood volume shift in the area of measurement causes an effect known as baseline drift. Changes in body position, muscle tremors, and electrode motions can also cause similar effects [62]. To model these effects, we consider these non-ECG components as either one of two components, i.e., multiplicative component α and additive component β, where those two components combine the rotation, scaling, baseline drift, and the additive Gaussian noise as shown in Equation (3),

(3) $$\tilde{x}\left(n\right)=\alpha \left(n\right)\cdot x\left(n\right)+\beta \left(n\right),$$

where $x,\;\tilde{x},\;\alpha ,\;\mathrm{and}\;\beta \in R^{N}$. The general aim would be to obtain the clean ECG signal *x* from the noisy ECG signal $\tilde{x}$ by suppressing the artifact components α and β. However, in this study, we are concerned with the additive component only, which represents the majority of different artifacts.

A denoising autoencoder (DAE) [32] utilizing self-organized Operational Neural Network (self-ONN) [48] layers is proposed to remove the additive artifacts in ECG signals. The DAE attempts to reconstruct the original clean data *x* from a corrupted version of these data $\tilde{x}$, usually by applying a stochastic corruption process $\tilde{x}∼q_{D}\left(\tilde{x}|x\right)$ such as applying Additive White Gaussian Noise (AWGN) or other means of partial destruction of the data. The encoder part of the DAE, $h=f_{\theta }\left(\tilde{x}\right)$, tries to map the corrupted input data to a lower-dimensional manifold while ignoring the variations of the input data. The decoder part of the DAE, $\hat{x}=g_{\theta }\left(h\right)$, then tries to reconstruct the clean data from the learned manifold. The overall DAE model can be summarized by Equation (4), where $\hat{x}\in R^{N}$ is the estimated clean data:

(4) $$\hat{x}=g_{\theta }\left(f_{\theta }\left(\tilde{x}\right)\right).$$

**Figure 1:**
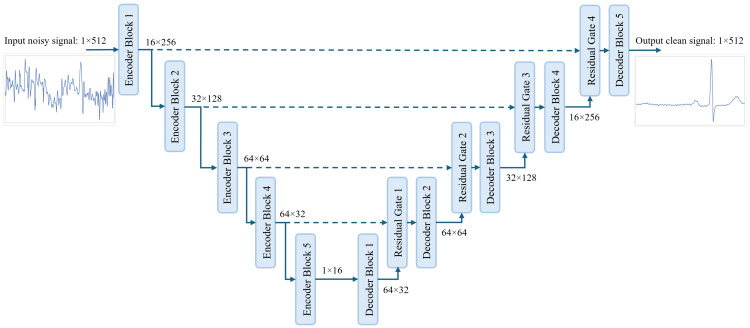
Overview of the proposed FGDAE model.

An overview of the proposed model is shown in Figure 1. In our proposed model we utilize the powerful U-Net architecture [57], which has proven to be efficient in ECG denoising. In U-Net architecture, the signal is passed through a few encoder layers that aim to find the latent representation of the input signal, which can be used by the decoder layers to reconstruct the clean ECG signal. The length of the signal after each encoder block is compressed, and the number of channels increases progressively. The opposite happens after each decoder layer, where the signal’s length increases and the number of channels decreases progressively. For the signal shape after each stage of the proposed model, we followed similar shapes as proposed by [36], which we found to be well suited for our model.

**Figure 2:**
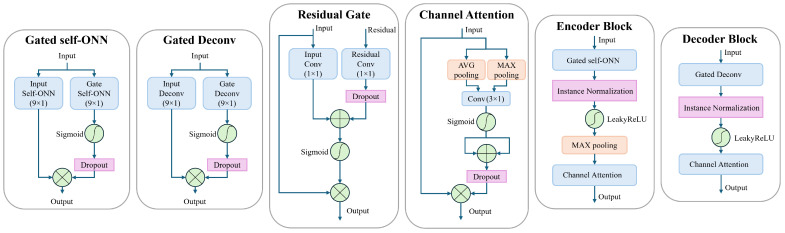
The building blocks of the proposed model.

The structures of the encoder and decoder blocks are shown in Figure 2. In the encoder block, we employ a gated self-ONN layer for feature extraction where two 1D self-ONN layers (kernel size = 9 × 1) parallelly process the input signal. One layer (input self-ONN) is extracting features from the input signal, while the other layer (gate self-ONN), followed by a sigmoid activation, is used to generate the gating mask to the extracted features. A dropout layer [63] is introduced to the gating path after the sigmoid activation to force the gate self-ONN to produce a robust gating mask and prevent overfitting, which is inspired by [64]. The dropout layer is by default active only during training, and we additionally limit it to be active only when the number of channels is more than 1 feature, which means that, for example, the dropout layer is not active in “Encoder block 5” shown in Figure 1. That also applies to all the dropout layers used anywhere in the proposed model. The gated self-ONN layer can be expressed by Equation (5),

(5) $$y=ONN_{i}\left(x\right)⊗\eta \left(\sigma \left(ONN_{g}\left(x\right)\right)\right),$$

where *x* and *y* are the input and output signals of the gated self-ONN layer, respectively. The ⊗ represents point-wise multiplication, $ONN_{i}\left(\cdot \right)$ and $ONN_{g}\left(\cdot \right)$ represent the input and gate self-ONN layers, respectively, generating input and gating feature maps, and σ(·) and η(·) represent sigmoid activation and dropout layers, respectively.

The decoder block uses a similar feature extraction layer structure (Gated Deconv) where the only difference is that the 1D self-ONN layers are replaced by 1D transpose convolutional (a.k.a. Deconvolution) layers (kernel size = 9 × 1). Equation (6) depicts the structure of the Gated Deconv block, where $Deconv_{i}\left(\cdot \right)$ and $Deconv_{g}\left(\cdot \right)$ represent the input and gate deconvolution layers, respectively, generating input and gating feature maps:

(6) $$y=Deconv_{i}\left(x\right)⊗\eta \left(\sigma \left(Deconv_{g}\left(x\right)\right)\right).$$

The feature extraction layers in encoder and decoder blocks are followed by a normalization layer where we employ the instance normalization [65], which in our experiments, seemed to give a better performance than batch normalization [66], for example. The instance normalization operates on each individual sample independently, rather than normalization across the entire batch as in the batch normalization. For non-linearity, we use LeakyReLU activation following the normalization layer. Then in the encoder only, a max pooling layer is utilized after the activation to divide the signal length in half as explained earlier. The decoder does not need a max-unpool layer since the stride is set to 2 in the “Gated Deconv” layers, which perform the needed unpooling effect. Finally, a channel attention module is utilized at the end of the encoder and decoder blocks. In our model, we use the efficient channel attention [35]. Yet, upon testing, we found that incorporating the information obtained from max pooling into the original average pooling resulted in a better performance. This is similar to the channel attention proposed by [37]. In the proposed model, we propose a small modification that enhances the performance of our model. Namely, the input signal is max-pooled and average-pooled simultaneously, the pooled features are passed through a shared 1D convolutional layer, and then each output is activated by a sigmoid activation function. The activation outputs are added together to form the attention mask. A dropout layer is applied to the attention mask to enhance generalization and prevent overfitting. The channel attention can be expressed as follows:

(7) $$\omega =\eta (\sigma \left(Conv\left(x_{max}\right)\right)+\sigma \left(Conv\left(x_{avg}\right)\right)),$$

(8) y=x⊗ω,

where *x* and *y* are the input and output signals of the channel attention block, respectively. The Conv(·) operator represents the shared 1D convolutional layer, and σ(·) and η(·) represent sigmoid activation and dropout layers, respectively. The attention mask is referred to as ω, while $x_{max}$ and $x_{avg}$ are the output features of applying global max pooling and global average pooling on the input signal, respectively. These are 1D features that have *C* data points, where *C* is the number of channels in the input signal *x*. We follow the other configurations for the convolutional layer proposed by [35], where, for the proposed model structure, the kernel size of the shared convolutional layer is 3 × 1, and “same padding” is used along all the used channel attention layers.

It is known that using residual connections, as in ResNet architectures [67], can help overcome issues like vanishing gradients and allows for the effective training of very deep networks. Recent works showed that residual connections in U-Net architectures have proven to be beneficial for ECG denoising. Furthermore, it has been shown by [62] that using spatial attention with point convolution on the residual connections on similar architecture helps in retaining the key features of the encoded feature maps and helps in better reconstruction in the decoder layers. Hence, in our work, we use the idea of residual gating proposed by Oktay et al. [68] to utilize the information from both the skip connection and the decoder output to form a gating for the decoder output as shown in Figure 2. The proposed residual gating is more global and robust than using attention on pooled features as in [62]. Both the spatial information and channel information are retained and utilized effectively to generate the required gating mask. In the proposed residual gating, two convolutional layers with a kernel size of 1 × 1 are used to generate features from the residual connection signal and the output of the preceding decoder block, respectively. A dropout layer is employed after the “Residual Conv” layer to put more emphasis on the output of the “Input Conv” layer while forcing more robust features from the residual connection. The rationale here is that the residual connection signal might still partially have noisy components, hence the need for more robustness. A sigmoid activation is used on the sum of the obtained features to generate the gating mask to the input signal of the residual gating block. The residual gate block can be described by Equation (9),

(9) $$y=x_{in}⊗\sigma \left(Conv_{in}\left(x_{in}\right)+\eta \left(Conv_{res}\left(x_{res}\right)\right)\right),$$

where $x_{in}$, $x_{res}$, and *y* are the input feature map to be gated (i.e., the output of the previous decoder block), the residual connection feature map, and the output of the residual gating block, respectively. The $Conv_{in}\left(\cdot \right)$ and $Conv_{res}\left(\cdot \right)$ operators represent the 1D convolutional layers (k = 1) that operate on input and residual feature maps, respectively. The σ(·) and η(·) operators, as usual, represent sigmoid activation and dropout layers, respectively.

### 3.2. Training Loss Functions

In this study, we aim to design a model that can benefit from the training process to gain the capability of performing effective denoising. Reducing the reconstruction error while keeping the morphology of the original signal as much as possible is of utmost priority. Hence, we designed a loss function that drives the model to learn the hidden features of the signal while retaining sparsity and generalization ability. As a reconstruction loss, we use the loss function proposed by [28] as shown in Equation (10),

(10) $$J_{recon}=\sum_{n=1}^{N}{\left[x\left(n\right)-\hat{x}\left(n\right)\right]}^{2}+\lambda \times \max|x\left(n\right)-\hat{x}\left(n\right){|}_{n=1}^{N},$$

where the sum of squared distance (SSD) (also defined in Equation (17)) and maximum absolute distance (MAD) (also defined in Equation (18)) are combined, and the tunable scaling coefficient λ for MAD is set to 50, similar to [28,36]. This combined loss ensures that the overall signal looks similar to the target signal while also minimizing the point-wise errors and rejecting outliers.

To enhance the spatial resolution of the reconstructed signal and encourage the model to pay attention to the fine details, a gradient loss (G-Loss) [69] is used as part of the overall model loss. The G-Loss was proposed by Ge et al. to improve the resolution of reconstructed images. In our case, it helps the model to follow the target signal closely and focus on subtle details that are not noise-related. The use of G-Loss also helps the model to learn a more detailed yet robust manifold of the ECG signals, which we believe would enhance the generalization ability of the model. We employed the idea of G-Loss, yet we calculated the MAD loss (instead of originally the sum of absolute error) between the gradients of the target signal *x* and the estimated signal $\hat{x}$. Following [69], we did not add a scaling parameter to this loss component. This loss component aims to minimize the largest deviation between the gradients, preventing outliers and localized high-intensity noise. Equations (11)–(13) depict the proposed gradient loss:

(11) $$x_{grad}\left(n\right)=x\left(n+1\right)-x\left(n\right),$$

(12) $${\hat{x}}_{grad}\left(n\right)=\hat{x}\left(n+1\right)-\hat{x}\left(n\right),$$

(13) $$J_{grad}=\max{\left|x_{grad}\left(n\right)-{\hat{x}}_{grad}\left(n\right)\right|}_{n=1}^{N-1}.$$

To further enhance the morphological similitude between the target signal and the estimated signal, we employ a correlation loss using Pearson’s correlation coefficient to measure the correlation between the signals. Exponential transformation of correlation values was used in [38] to enhance the correlation between power spectrums of the target and the estimated signals, while the absolute of the correlation was used as auxiliary loss in [70] to enhance the morphology of ECG generated from given photoplethysmography (PPG) signals. In this study, we wanted to leverage the overall morphological resemblance between the target and the estimated signals, and hence we penalize the minimum correlation as shown in Equation (14):

(14) $$J_{morph}=\lambda_{morph}\times \left(1-\min\left(\frac{\mathrm{cov}(x_{i},{\hat{x}}_{i})}{\sigma_{x_{i}}\sigma_{{\hat{x}}_{i}}}\right)\right).$$

The rationale here is that the minimum correlations usually come from outliers or difficult pathological cases, and we wanted our model to be able to retain the morphology even for these challenging samples. The correlation loss is computed as $1-min(correlation\left(x_{i},{\hat{x}}_{i}\right))$, where $x_{i}$ and ${\hat{x}}_{i}$ are the $i^{th}$ target and estimated samples. This means that the possible range of this loss component is from 0 to 2. The tunable scaling factor $\lambda_{morph}$ is used to balance the significance of this component to other components of the overall loss. Empirically, we tested the values of $\lambda_{morph}$ in the range of 5 to 30 and found 10 to be suitable for our model, where it is neither too large to dominate other loss components nor small enough to be negligible.

To promote the model’s sparsity, we employ $L_{1}$ regularization on the weights of the encoder part of the model to ensure sparse and robust manifold learning. L1 regularization adds a penalty equal to the absolute value of the magnitude of the coefficients. L1 regularization can lead to sparse models where some weights become exactly zero, effectively selecting features and preventing overfitting [71]. The $L_{1}$-regularization cost function ($J_{reg}$) is described in Equation (15),

(15) $$J_{reg}=\lambda_{reg}\sum_{p}\left|\theta_{p}^{Enc}\right|,$$

where $\theta_{p}^{Enc}$ is the set of weights of the $p^{th}$ layer of the encoder, while the tunable weight decay factor $\lambda_{reg}$ is used to control the strength of regularization. Empirically, we tested values for $\lambda_{reg}$ in the range of 0.005 to 1 and found that 0.01 was suitable for our model. We noticed that higher values of $\lambda_{reg}$ negatively affect the model performance and prevent it from converging due to over-regularization. A very low value of $\lambda_{reg}$ makes the loss value very small, to a limit that it doesn’t affect the performance of the model.

The overall loss function ($J_{AE}\left(\theta \right)$) is the sum of all the discussed terms as shown in Equation (16), promoting better reconstruction, morphological similitude, and sparsity:

(16) $$J_{AE}\left(\theta \right)=J_{recon}+J_{grad}+J_{morph}+J_{reg}.$$

### 3.3. Datasets and Experimental Setup

In this study, we follow the benchmark experiment guidelines proposed by [28]. The benchmark tests the model performance on a dataset created by combining signals from two openly available databases: QT Database (QTDB) [72] and the MIT-BIH Stress Test Database (NSTDB) [73]. The QTDB provides the clean ECG samples collected from 7 other databases. A total of 105 records of dual-channel ECG with a duration of 15 min each with a sampling rate of 250 Hz are available from this database collected from different leads and containing different pathological cases. The NSTDB provides three artifact types (namely, baseline wanders (BW), muscle artifacts (MA), and electrode motions (EM)) that resemble ambulatory ECG noise. Each available artifact type has a duration of 30 min and a sampling rate of 360 Hz. The samples from QTDB are resampled to 360 Hz to match the sampling rate of noise records. In the original benchmark experiment, the ECG records from the QT database are corrupted by the noise in NSTDB that contains baseline wander (BW) only. The contamination noise samples are scaled randomly to have maximum amplitudes in the range [0.2:2] times the ECG sample maximum amplitude. Since NSTDB has noise records in two channels, each channel is split in half (where the first and second halves are referred to as “a” and “b”, respectively), and one half from each channel is used for either training or testing as shown in Table 1.

The selected models are trained on ECG signals corrupted with “channel 1_a” and tested on ECG signals corrupted with “channel 2_b” (this noise version is referred to as “Noise v1 (nv1)” elsewhere in this study) and then trained on ECG signals corrupted with “channel 2_a” and tested on ECG signals corrupted with “channel 1_b” (this noise version is referred to as “Noise v2 (nv2)” elsewhere in this study). This ensures that each time the model is trained and tested on different uncorrelated noises. The combined results of the two experiments are then reported, as in the following section. The test datasets use selected subjects from the QT database as shown in Table 2. The selected test set represents 13% of the total data amount. Using that inter-patient scheme for data division ensures better evaluation of the model’s generalization capability and adapts to real-world scenarios.

However, the original experiment assumes non-realistic assumptions for the data preparation, such as having one beat per signal (data sample) centered with zero-padding to ensure size 512 per data sample. In most real-life situations it is not possible to perform that segmentation process before denoising, especially when the signal is too corrupted. Furthermore, assuming spatial consistency in the input samples is impractical. Also, using only one noise type as in [28] or all three noise types combined as in [36] does not cover the possibilities of real noisy signals. Hence, we follow the proposed data preparation scheme proposed by [38], where a sliding window of length 512 and overlap of 256 is used to segment the signals into data samples of length 512 each with no prior assumptions of the beat length or its spatial position in the obtained signal. Additionally, we noticed that the clean ECG signals are not entirely clean, as some of the segments include very slow baseline variations. We removed these variations by subtracting the mean of the segments. Following [28], no amplitude normalizations of the clean ECG signals were done. Furthermore, we utilize the same Random Mixed Noise (RMN) scheme proposed by [38], where 8 random combinations of noise are created in the training and testing datasets (shown in Table 3), giving more variation to the datasets with more realistic scenarios.

The random noise scaling and random noise type combinations are independent of each other. An important point in this combination is that it provides clean signal samples, which is essential to have in the training datasets for the deep learning models to influence the denoising functionality only on noisy signals rather than blindly performing reduction in innate features of the signal. For each noise split, we end up with 91,062 pairs of noisy (input) and clean (reference) ECG samples for training data and 15,535 pairs for testing data.

### 3.4. Comparative Methods

We compare our proposed algorithm with different methods, including state-of-the-art methods. These methods can be divided into three categories:

#### 3.4.1. Traditional Filters

We use FIR and IIR filters as non-learnable baseline methods as in previous studies employing this benchmark setup. The implementation of the 4th order Butterworth IIR filter is more computationally efficient, yet both filters are bidirectional (zero-phase) high-pass filters with a cut-off frequency of 0.67 Hz. The design of both the IIR and the FIR filters is described in [28].

#### 3.4.2. Non-DAEs

Additionally, we compare with deep learning methods that do not compress the input signals to lower dimensions, such as in DAEs. For this category, we use the same methods used in previous state-of-the-art studies, such as deep recurrent neural networks (DRNNs) proposed by [27] and a multi-branch deep convolutional filter model (DeepFilter) proposed by [28].

#### 3.4.3. DAEs

We compare our model with similar DAE-based models that include the state-of-the-art methods such as FCN-DAE [33], ACDAE [34], CBAM-DAE [36], and TCDAE [38]. We also include a convolutional denoising autoencoder (CNN-DAE) model adopted in the experimentation of [36]. The CNN-DAE model is very similar to FCN-DAE, except that the output transpose convolution layer is replaced by a dense (fully connected) layer in CNN-DAE. For the ablation study, we also include our proposed model with different values of the “*q*” parameter of self-ONN to evaluate the behavior of the model for each value. For q=1, the model has no non-linear layers (typical of traditional “Conv” layers), and non-linearity is introduced gradually as the set value to the “*q*” parameter increases. The dropout ratio is set to 0.001×q for the gated self-ONN layer and 0.001 otherwise in other layers in the model.

### 3.5. Evaluation Metrics

To evaluate our model’s performance compared with other methods, we use the same four quantitative evaluation metrics used in [28] as well as another three standard error metrics used in the literature to evaluate signal denoising effectiveness. The following is a list of the seven used evaluation metrics:

1. Sum of the square of the distances (SSD)
  (17) $$\mathrm{SSD}=\sum_{n=1}^{N}{\left[x\left(n\right)-\hat{x}\left(n\right)\right]}^{2}$$
2. Maximum absolute distance (MAD)
  (18) $$\mathrm{MAD}=\max{\left|x\left(n\right)-\hat{x}\left(n\right)\right|}_{n=1}^{N}$$
3. Percentage root-mean-square difference (PRD)
  (19) $$\mathrm{PRD}=\sqrt{\frac{\sum_{n=1}^{N}{\left[x\left(n\right)-\hat{x}\left(n\right)\right]}^{2}}{\sum_{n=1}^{N}{\left[x\left(n\right)-\bar{x}\right]}^{2}}}\times 100\%$$
4. Cosine similarity (CosSim)
  (20) $$\mathrm{CosSim}=\frac{\sum_{n=1}^{N}x\left(n\right)\hat{x}\left(n\right)}{\sqrt{\sum_{n=1}^{N}{\left[x\left(n\right)\right]}^{2}}\sqrt{\sum_{n=1}^{N}{\left[\hat{x}\left(n\right)\right]}^{2}}}$$
5. Mean absolute error (MAE)
  (21) $$\mathrm{MAE}=\frac{1}{N}\sum_{n=1}^{N}\left|x\left(n\right)-\hat{x}\left(n\right)\right|$$
6. Root mean square error (RMSE)
  (22) $$\mathrm{RMSE}=\sqrt{\frac{1}{N}\sum_{n=1}^{N}{\left[x\left(n\right)-\hat{x}\left(n\right)\right]}^{2}}$$
7. Signal-to-noise ratio (SNR)
  (23) $$\mathrm{SNR}=10\log_{10}\left(\frac{\sum_{n=1}^{N}{\left[x\left(n\right)\right]}^{2}}{\sum_{n=1}^{N}{\left[x\left(n\right)-\hat{x}\left(n\right)\right]}^{2}}\right)$$

where *N* is the number of samples in the signals, *x* and $\hat{x}$ are original (clean) and reconstructed (denoised) signals, respectively, and $\bar{x}$ is the mean of the original signal. These different quantitative measures indicate the performance of the models from various perspectives. For instance, SSD and RMSE evaluate the error in denoising while giving more weight to the larger errors. MAD identifies extreme outliers, while PRD provides normalized overall error measures accounting for different signal segment magnitudes. MAE provides a straightforward interpretation of the average error without excessive sensitivity to outliers. SNR indicates how well the noise component is suppressed compared with the signal component. Finally, cosine similarity measures the preservation of signal shape and structure, independent of amplitude errors.

### 3.6. Training and Implementation Details

The proposed model and other comparative models were implemented in Python using the Tensorflow Keras framework (The implementation codes will be available at https://github.com/AhmedAShaheen/fully_gated_DAE (accessed on 26 January 2025)). The core of the benchmark is obtained from [28] and other additions provided by [36,38]. All the methods were trained and tested on the same benchmark using the same conditions. The deep learning models were trained and tested on GPU, and traditional filters were tested on CPU on a regular laptop with an AMD Ryzen 7 5800H CPU, 32 GB RAM, and NVIDIA GeForce RTX 3080 GPU. A batch size of 64 was used for training, as well as using the Adam optimizer with an initial learning rate of $10^{-3}$. Similar to [28], we reduce the learning rate by a factor of 2 after a couple of iterations if the validation loss does not improve by a minimum of 0.05. The minimum possible learning rate was set to $10^{-10}$ as well. The training was permitted for $10^{5}$ iterations unless the validation loss stops improving for 10 consecutive iterations. The weights resulting in the best validation loss for each noise version for each model were saved and used in the evaluation process.

## 4. Results

In this section, we present the proposed model’s performance results compared with other comparative models in the benchmark. Following [38], we also used the Wilcoxon signed-rank test to compare the outputs of all methods with the proposed model. The significance level for the test (α) is also set to 0.05.

### 4.1. Overall Quantitative Evaluation

The overall error metrics for the tested denoising methods are shown in Table 4, where the MEAN and standard deviation (STD) values (MEAN±STD) are reported for each metric for each method. The MEAN and STD values are calculated from the error between denoised and reference segments in the testing data for both noise versions (concatenated). The statistical significance (*p*-value) is reported for other models compared with FGDAE for (q=1) and (q=2). The results show superior overall performance of the FGDAE model (q=2) compared with all other methods except for PRD and SNR, where the FGDAE model (q=1) shows better performance. The SSD, MAD, PRD, CosSim, RMSE, MAE, and SNR of FGDAE (q=2) are 12.251±30.766 mV, 0.501±0.448 mV, 50.588±31.524%, 0.867±0.165, 0.121±0.096 mV, 0.086±0.065 mV, and 7.841±4.749 dB, respectively. Likewise, the SSD, MAD, PRD, CosSim, RMSE, MAE, and SNR of FGDAE (q=1) are 12.899±32.987 mV, 0.504±0.466 mV, 49.993±30.778%, 0.866±0.169, 0.122±0.102 mV, 0.086±0.068 mV, and 8.028±5.229 dB, respectively. However, the overall performance difference between the FGDAE model (q=1) and the FGDAE model (q=2) is significant in favor of (q=2). The results propose that the additional increase in non-linearity level can be unpleasant and result in worse performance for the proposed model design, as in cases of (q=3) and (q=4). Hence, in the following sections, we only report the results of the two best models, i.e., FGDAE with q=1 and q=2. Henceforth, we refer to those models as “linear” and “non-linear” FGDAE, respectively.

The statistical significances per error metric for selected methods (the closest performance to our model) compared with the proposed non-linear FGDAE model are shown in Table 5. It can be seen from the results that the *p*-values for all the selected models are almost zero, indicating significant performance of the proposed non-linear FGDAE. The results also show that the PRD and SNR differences between the linear and non-linear FGDAE models are significant. This indicates that the level of non-linearity can be adjusted in the model to fulfill specific needs.

### 4.2. Performance Under Different Noise Levels

We calculate the error metrics for different noise amplitudes for each method similar to the analysis proposed by [28] to evaluate the performance of the models in different noise severities. Table 6, Table 7, Table 8, Table 9, Table 10, Table 11 and Table 12 show the results of the used metrics separately. The *p*-values are calculated in these tables for all models compared with the best-performing model on the specified scales. The SSD results in Table 6 show that the non-linear FGDAE model performs best (with statistical significance) on the noisiest signals, i.e., ranges (1.0to1.5) and (1.5to2.0). Although the linear FGDAE produces better results for ranges (0.2to0.6) and (0.6to1.0), the statistical difference in performance is not significant in range (0.6to1.0). Similar results can be seen in Table 7, Table 10, and Table 11 for MAD, RMSE, and MAE, respectively. Table 8 shows that linear FGDAE shows the best PRD error values in ranges (0.2to0.6), (1.0to1.5), and (1.5to2.0), while non-linear FGDAE shows the best values in the range (0.6to1.0). However, the differences in ranges (0.2to0.6), (1.0to1.5), and (1.5to2.0) are not significant, showing that the performance w.r.t. that metric on selected noise amplitudes is comparable for both models. For CosSim results shown in Table 9, it can be noticed that the linear model performs best in the lowest range (0.2to0.6) while the non-linear model performs best otherwise. The statistical difference in the range (0.6to1.0) is not significant, though. This is very similar to the SNR results reported in Table 12. These results clearly indicate that the non-linear model preserves the morphological similarity better than all other models, especially in cases of larger noise amplitudes. This can be attributed to the fact that the introduced non-linearity tends to learn more complex features, making it more suitable for processing more complex signals.

### 4.3. Performance Under Different Noise Types

To evaluate the model’s robustness on different noises, we calculated the error metrics for different noise types. For brevity, we only compared our methods’ results to the results of the state-of-the-art model (i.e., TCDAE) present in Table 13. Table 14 and Table 15 show the results for the linear and non-linear FGDAE models, respectively. The results show that the linear FGDAE outperforms the non-linear model as well as TCDAE when applied to clean signals. The non-linear FGDAE becomes marginally inferior to TCDAE only on clean ECG signals; otherwise, both linear and non-linear FGDAE show better performance compared with TCDAE on all artifact compositions. The non-linear model seems to introduce more disturbances to clean signals, which can be attributed to the possibility that the non-linear model is biased more towards denoising more complex signals than cleaner ones. The results also show that BW and MA artifacts and their combinations seem to be less problematic than EM for the three models. It can be noticed that the three models produce significantly more error for EM and other combinations that include EM than any other noise combinations. The electrode motion noise is expected to be very challenging generally since it introduces very sharp drops or severe distortion in the data compared with motion and baseline wander artifacts, which have naturally slower trends. Nonetheless, the electrode motion is very difficult to model compared with the other two noise types. However, especially in these challenging cases, the non-linear model outperforms the linear model and TCDAE. This shows that although the non-linear model can be a bit problematic for less noisy data, it can introduce major benefits in denoising more challenging data.

### 4.4. Visual Analysis of Denoising Performance

**Figure 3:**
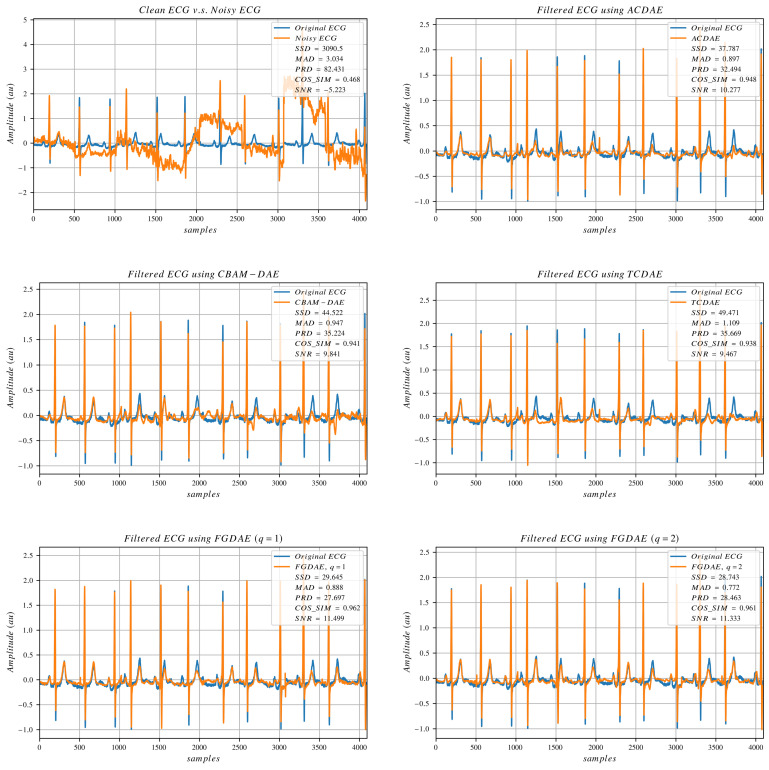
ECG signals processed by selected algorithms. The artifact composition is (BW+MA).

**Figure 4:**
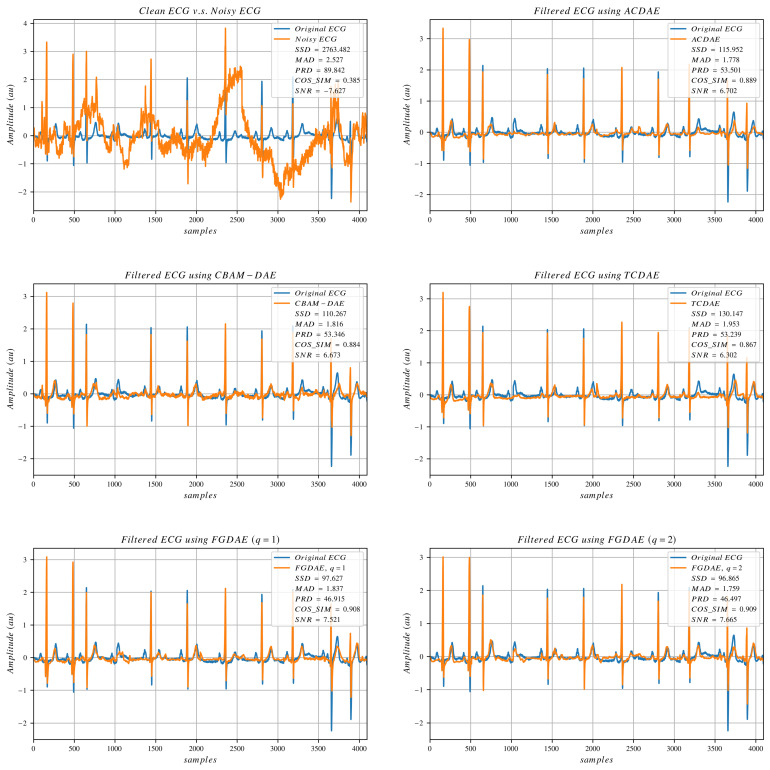
ECG signals processed by selected algorithms. The artifact composition is (MA+EM).

**Figure 5:**
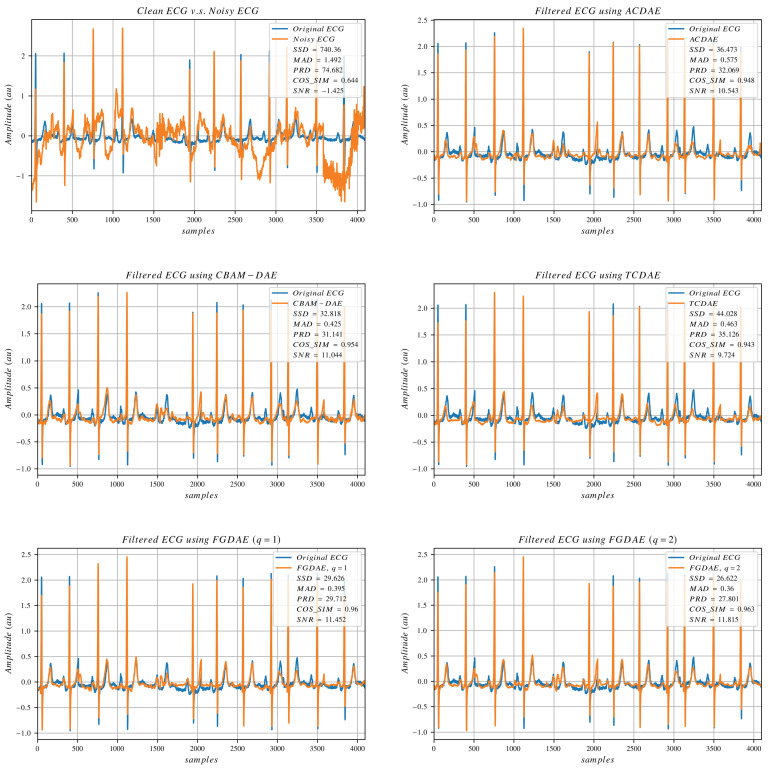
ECG signals processed by selected algorithms. The artifact composition is (BW+MA+EM).

To visually evaluate the denoising performance of different algorithms, we plot a small portion of the data, making a total of 4069 data points (8 consecutive non-overlapping segments), which is a bit more than 11 s per plot. Figure 3, Figure 4 and Figure 5 show signals collected from different subjects, with different artifact compositions and different noise amplitudes. The clean and noisy signals are displayed in the top-left plot of the three figures, accompanied by SNR and other error metrics calculated between the clean and noisy signals. The denoised outputs of selected state-of-the-art models, along with our linear and non-linear FGDAE models, are also presented in the same figures. The figures demonstrate that the linear FGDAE produces the best-preserved signals in the case of BW+MA, while the non-linear FGDAE model achieves better results in the other two cases involving EM artifacts. This outcome is consistent with the findings presented in the previous section. Although the differences between the linear and non-linear models appear marginal, the plots indicate that the SNR improvement achieved by both models is considerable compared with other methods. The figures demonstrate that both FGDAE models effectively preserve the key morphological features of the ECG signal, even in abnormal cases. Compared with other state-of-the-art methods, they exhibit less distortion and smaller deviations relative to the reference clean signal. However, in our models, some P and T waves still fail to align sufficiently well. These minor disturbances and mismatches could potentially lead to incorrect diagnoses. Thus, while the proposed solution represents a considerable improvement, perfect morphological preservation of ECG signals in these situations is not guaranteed, highlighting the need for further improvements.

### 4.5. Computational Efficiency

Finally, we summarize the model sizes and inference time for testing data for both noise versions (i.e., nv1 and nv2) for selected models in Table 16. The proposed linear and non-linear models have 263.25k and 384.50k parameters, respectively. Compared with the TCDAE model with over 1 million parameters, our proposed models show a significant reduction of 61% to 73%. This comes with comparable inference time for both models. We believe that the gating mechanisms in the self-ONN layers and the residual connections introduce some extra delay in the model, causing it to have a similar inference time to the TCDAE model despite the smaller sizes of the proposed models. Also, the TCDAE has fewer stages, yet there is a large bottleneck at the transformer part where many attention heads work concurrently. Since we are using a GPU for training and testing, the provided parallelism causes the TCDAE to have close inference times to the proposed models. Also, we believe that the non-linear version of the proposed model could be optimized to be more compact, yet for the sake of fair comparison, we keep the model structure as it is for the linear and non-linear versions, where the linear version is the baseline for designing the model architecture. However, as aforementioned, the compact self-ONN models can in fact surpass more complex CNN models if carefully designed.

## 5. Conclusions

An efficient model is proposed in this study to reduce different types of artifacts in ECG signals. The proposed FGDAE model employs the U-Net architecture for DAE to perform a robust denoising process. FGDAE utilized the idea of gating and attention efficiently to provide a high denoising performance. The linear and non-linear variants of the proposed model harness the feature extraction capability of CNN and self-ONNs, respectively, to achieve state-of-the-art ECG denoising performance in all possible noise combinations and amplitudes compared with the baseline methods in the followed benchmark. The seven used evaluation metrics show that the proposed model with its linear and non-linear variants shows statistically significant performance differences compared with the other methods in the benchmark. The proposed model succeeds in preserving the key features of the ECG signals, such as QRS complexes and P and T waves, in most cases, being superior to other baseline models, especially in intensive noise contamination. The proposed model size is significantly smaller compared with the TCDAE model and has a comparable inference time. The provided performance shows that the FGDAE model can be used in the vast majority of noisy ECG conditions to generate a high-quality denoised signal. The results show that utilizing gating mechanisms in the proposed structure indeed provides significant value. Also, the results show proof of concept for the great potential of introducing non-linearities in the denoising architecture.

A clear limitation of our model is the elimination of EM artifacts. The abrupt large changes and the spikes that look very similar to morphological ECG features in that artifact make it challenging to remove. That limitation is important to investigate in future works to enable more reliable ECG denoising solutions. In future works, we intend to customize the proposed model more to unlock the full power of the self-ONN layers with more non-linearity. We also plan to employ multimodalities such as synchronized motion signals to ensure better signal processing. Utilizing motion references would give a more reliable indication of the type and structure of the contaminating motion. Also, it can provide objective information on the quality of the ECG signal, which would be beneficial in rejecting heavily corrupted ECG segments. Finally, to validate the usefulness of the proposed model, we intend to perform diagnostic tasks where the morphology preservation of the signal is critical.

## Figures and Tables

**Table 1:** Noise split used in the experiments.

Split Name	Train Noise	Test Noise
Noise split version 1 (nv1)	Channel 1_a	Channel 2_b
Noise split version 2 (nv2)	Channel 2_a	Channel 1_b

**Table 2:** Subjects selected from QTDB for test sets.

Database Name	Selected ID
MIT-BIH Arrhythmia Database	sel123, sel233
MIT-BIH ST Change Database	sel302, sel307
MIT-BIH Supraventricular Arrhythmia Database	sel820, sel853
MIT-BIH Normal Sinus Rhythm Database	sel16420, sel16795
European ST-T Database	sel0106, sel0121
Sudden death patients from BIH	sel32, sel49
MIT-BIH Long-Term ECG Database	sel14046, sel15815

**Table 3:** Noise types and combination types.

Noise Type *	Combination Type							
	1	2	3	4	5	6	7	8
BW	-	✔	-	✔	-	✔	-	✔
MA	-	-	✔	✔	-	-	✔	✔
EM	-	-	-	-	✔	✔	✔	✔

* The symbol "-" represents the absence of a noise type and the symbol "✔" indicates the presence of a noise type in each combination.

**Table 4:** Overall evaluation metrics results for different methods for RMN experiment.

Method *	SSD (mV)	MAD (mV)	PRD (%)	CosSim (a.u.)	RMSE (mV)	MAE (mV)	SNR (dB)	*p*-Value (q = 1)	*p*-Value (q = 2)
FIR Filter [28]	85.705±153.683	0.959±0.861	64.552±27.458	0.685±0.238	0.181±0.119	0.127±0.079	4.162±4.290	0.000	0.000
IIR Filter [28]	87.466±152.595	1.012±0.876	67.597±25.297	0.667±0.235	0.181±0.119	0.127±0.079	4.162±4.290	0.000	0.000
DRNN [27]	25.711±45.872	0.831±0.619	89.806±51.404	0.720±0.235	0.182±0.131	0.121±0.079	4.127±4.271	0.000	0.000
CNN-DAE [36]	20.654±29.592	0.913±0.528	98.721±42.564	0.754±0.200	0.173±0.102	0.112±0.068	4.044±2.707	0.000	0.000
DeepFilter [28]	18.011±34.857	0.616±0.494	66.110±33.461	0.811±0.174	0.152±0.110	0.108±0.070	5.679±4.349	0.000	0.000
FCN-DAE [33]	17.737±35.363	0.707±0.539	68.630±38.680	0.803±0.201	0.151±0.109	0.103±0.071	5.629±3.938	0.000	0.000
ACDAE [34]	13.715±31.486	0.570±0.477	54.561±32.317	0.850±0.174	0.129±0.101	0.089±0.066	7.300±4.824	0.000	0.000
CBAM-DAE [36]	16.332±35.659	0.561±0.494	54.284±29.688	0.837±0.189	0.138±0.114	0.098±0.076	6.916±5.102	0.000	0.000
TCDAE [38]	15.399±34.832	0.580±0.495	52.632±32.179	0.850±0.187	0.134±0.110	0.093±0.074	7.183±5.261	0.000	0.000
FGDAE, q = 1 (ours)	12.899±32.987	0.504±0.466	49.993±30.778	0.866±0.169	0.122±0.102	0.086±0.068	8.028±5.229	−	$8.58\times 10^{-13}$
FGDAE, q = 2 (ours)	12.251±30.766	0.501±0.448	50.588±31.524	0.867±0.165	0.121±0.096	0.086±0.065	7.841±4.749	$8.58\times 10^{-13}$	−
FGDAE, q = 3 (ours)	14.520±32.859	0.563±0.480	55.960±32.964	0.847±0.171	0.133±0.104	0.093±0.070	7.119±4.925	0.000	0.000
FGDAE, q = 4 (ours)	24.100±34.926	0.775±0.576	80.954±43.806	0.738±0.210	0.181±0.119	0.127±0.079	4.162±4.290	0.000	0.000

* The bold black values indicate the best performance for each error metric.

**Table 5:** Wilcoxon test (α=0.05) results between (FGDAE, q = 2) and selected models.

Metric	ACDAE [34]	CBAM-DAE [36]	TCDAE [38]	FGDAE, q = 1
SSD	0.0	0.0	0.0	0.0152
MAD	0.0	0.0	0.0	$4.77\times 10^{-5}$
PRD	0.0	0.0	$2.76\times 10^{-135}$	$3.99\times 10^{-15}$
CosSim	0.0	0.0	$8.21\times 10^{-174}$	0.002
RMSE	0.0	0.0	0.0	$5.94\times 10^{-10}$
MAE	$6.40\times 10^{-207}$	0.0	$8.79\times 10^{-272}$	$7.08\times 10^{-17}$
SNR	0.0	0.0	0.0	$4.88\times 10^{-39}$

**Table 6:** SSD in different noise segments for different methods for RMN experiment.

Method *	0.2to0.6	*p*-Value	0.6to1.0	*p*-Value	1.0to1.5	*p*-Value	1.5to2.0	*p*-Value
FIR Filter [28]	13.281±18.963	0.000	45.027±51.702	0.000	108.33±133.80	0.000	201.64±235.83	0.000
IIR Filter [28]	19.914±36.081	0.000	48.996±61.473	0.000	109.61±138.85	0.000	194.72±231.93	0.000
DRNN [27]	9.540±11.546	0.000	17.249±19.915	0.000	31.911±44.748	0.000	51.011±72.185	0.000
CNN-DAE [36]	13.153±14.126	0.000	15.958±16.146	0.000	22.618±27.114	0.000	33.395±47.069	0.000
DeepFilter [28]	6.773±8.176	0.000	12.130±13.265	0.000	22.019±30.671	0.000	35.997±58.169	0.000
FCN-DAE [33]	7.739±10.043	0.000	11.218±13.435	0.000	19.732±29.973	0.000	35.475±59.870	0.000
ACDAE [34]	4.842±7.299	0.000	8.428±10.438	0.000	15.785±23.642	0.000	29.279±55.296	0.000
CBAM-DAE [36]	4.790±6.770	0.000	9.415±12.309	0.000	19.425±29.351	0.000	36.018±60.538	0.000
TCDAE [38]	5.034±8.310	0.000	8.976±12.185	0.000	18.079±29.706	0.000	33.436±59.043	0.000
FGDAE, q = 1	4.298±7.116	–	7.405±10.228	–	14.599±25.467	0.000	28.665±58.270	0.000
FGDAE, q = 2	4.431±7.571	0.000	7.477±10.904	0.161	13.778±23.923	–	26.230±54.206	–
FGDAE, q = 3	4.962±7.444	0.000	8.829±10.827	0.000	16.747±26.402	0.000	31.547±56.763	0.000
FGDAE, q = 4	9.961±12.276	0.000	17.620±18.185	0.000	30.706±36.328	0.000	44.672±50.174	0.000

* The bold black values indicate the best performance for each noise amplitude range.

**Table 7:** MAD in different noise segments for different methods for RMN experiment.

Method *	0.2to0.6	*p*-Value	0.6to1.0	*p*-Value	1.0to1.5	*p*-Value	1.5to2.0	*p*-Value
FIR Filter [28]	0.415±0.267	0.000	0.800±0.465	0.000	1.243±0.748	0.000	1.716±0.999	0.000
IIR Filter [28]	0.505±0.401	0.000	0.846±0.532	0.000	1.268±0.790	0.000	1.707±1.033	0.000
DRNN [27]	0.525±0.360	0.000	0.742±0.453	0.000	0.991±0.612	0.000	1.231±0.731	0.000
CNN-DAE [36]	0.791±0.440	0.000	0.858±0.461	0.000	0.968±0.551	0.000	1.078±0.618	0.000
DeepFilter [28]	0.391±0.253	0.000	0.529±0.317	0.000	0.727±0.493	0.000	0.924±0.645	0.000
FCN-DAE [33]	0.494±0.320	0.000	0.612±0.384	0.000	0.799±0.553	0.000	1.008±0.692	0.000
ACDAE [34]	0.352±0.246	0.000	0.479±0.301	0.000	0.666±0.461	0.000	0.884±0.626	0.000
CBAM-DAE [36]	0.318±0.213	0.000	0.466±0.293	0.000	0.672±0.479	0.000	0.908±0.651	0.000
TCDAE [38]	0.343±0.237	0.000	0.469±0.295	0.000	0.684±0.480	0.000	0.922±0.653	0.000
FGDAE, q = 1	0.289±0.206	–	0.415±0.260	–	0.600±0.445	0.000	0.834±0.626	0.000
FGDAE, q = 2	0.292±0.202	0.026	0.416±0.266	0.461	0.589±0.431	–	0.810±0.602	–
FGDAE, q = 3	0.331±0.221	0.000	0.476±0.293	0.000	0.667±0.470	0.000	0.905±0.614	0.000
FGDAE, q = 4	0.497±0.353	0.000	0.687±0.425	0.000	0.931±0.580	0.000	1.146±0.662	0.000

* The bold black values indicate the best performance for each noise amplitude range.

**Table 8:** PRD in different noise segments for different methods for RMN experiment.

Method *	0.2to0.6	*p*-Value	0.6to1.0	*p*-Value	1.0to1.5	*p*-Value	1.5to2.0	*p*-Value
FIR Filter [28]	45.648±17.702	0.000	67.590±16.844	0.000	79.625±15.107	0.000	87.113±12.259	0.000
IIR Filter [28]	51.004±18.884	0.000	69.377±17.036	0.000	80.230±15.564	0.000	86.882±13.269	0.000
DRNN [27]	64.790±34.190	0.000	88.955±47.679	0.000	107.07±51.57	0.000	117.82±50.34	0.000
CNN-DAE [36]	84.010±29.592	0.000	94.703±37.856	0.000	107.36±46.60	0.000	116.34±49.22	0.000
DeepFilter [28]	49.299±22.593	0.000	63.670±27.485	0.000	77.371±31.730	0.000	87.835±33.286	0.000
FCN-DAE [33]	52.159±26.203	0.000	63.721±33.709	0.000	77.275±40.100	0.000	89.844±42.912	0.000
ACDAE [34]	37.672±17.800	0.000	50.834±24.278	0.000	64.697±31.349	0.000	77.128±35.294	0.000
CBAM-DAE [36]	37.723±16.918	0.000	51.342±22.496	0.000	64.450±28.223	0.000	75.512±30.700	0.000
TCDAE [38]	38.300±19.313	0.000	48.872±24.088	0.000	61.278±32.155	0.000	72.827±36.759	0.000
FGDAE, q = 1	34.699±18.301	–	46.950±23.058	0.032	59.418±29.317	–	71.299±32.849	–
FGDAE, q = 2	34.832±18.713	0.250	46.607±23.760	–	59.504±30.628	0.649	71.343±35.582	0.860
FGDAE, q = 3	37.858±18.253	0.000	52.842±24.227	0.000	67.487±30.683	0.000	79.904±34.180	0.000
FGDAE, q = 4	55.685±27.803	0.000	78.073±32.848	0.000	97.789±40.121	0.000	110.78±43.65	0.000

* The bold black values indicate the best performance for each noise amplitude range.

**Table 9:** CosSim in different noise segments for different methods for RMN experiment.

Method *	0.2to0.6	*p*-Value	0.6to1.0	*p*-Value	1.0to1.5	*p*-Value	1.5to2.0	*p*-Value
FIR Filter [28]	0.875±0.095	0.000	0.715±0.149	0.000	0.575±0.184	0.000	0.460±0.196	0.000
IIR Filter [28]	0.841±0.128	0.000	0.697±0.159	0.000	0.566±0.187	0.000	0.461±0.200	0.000
DRNN [27]	0.875±0.086	0.000	0.769±0.151	0.000	0.638±0.216	0.000	0.511±0.246	0.000
CNN-DAE [36]	0.854±0.073	0.000	0.802±0.121	0.000	0.713±0.197	0.000	0.608±0.264	0.000
DeepFilter [28]	0.910±0.060	0.000	0.845±0.107	0.000	0.761±0.166	0.000	0.672±0.211	0.000
FCN-DAE [33]	0.903±0.066	0.000	0.853±0.118	0.000	0.764±0.198	0.000	0.652±0.267	0.000
ACDAE [34]	0.942±0.045	0.000	0.890±0.096	0.000	0.811±0.166	0.000	0.714±0.231	0.000
CBAM-DAE [36]	0.941±0.048	0.000	0.879±0.106	0.000	0.790±0.181	0.000	0.688±0.246	0.000
TCDAE [38]	0.941±0.055	0.000	0.890±0.105	0.000	0.810±0.183	0.000	0.714±0.251	0.000
FGDAE, q = 1	0.950±0.044	–	0.905±0.088	0.076	0.832±0.158	0.001	0.735±0.232	0.000
FGDAE, q = 2	0.949±0.045	0.005	0.906±0.088	–	0.835±0.158	–	0.741±0.227	–
FGDAE, q = 3	0.943±0.043	0.000	0.890±0.088	0.000	0.808±0.156	0.000	0.701±0.222	0.000
FGDAE, q = 4	0.896±0.064	0.000	0.790±0.118	0.000	0.658±0.173	0.000	0.528±0.204	0.000

* The bold black values indicate the best performance for each noise amplitude range.

**Table 10:** RMSE in different noise segments for different methods for RMN experiment.

Method *	0.2to0.6	*p*-Value	0.6to1.0	*p*-Value	1.0to1.5	*p*-Value	1.5to2.0	*p*-Value
FIR Filter [28]	0.121±0.069	0.000	0.166±0.083	0.000	0.216±0.115	0.000	0.261±0.138	0.000
IIR Filter [28]	0.121±0.069	0.000	0.166±0.083	0.000	0.216±0.115	0.000	0.261±0.138	0.000
DRNN [27]	0.121±0.062	0.000	0.163±0.084	0.000	0.214±0.129	0.000	0.266±0.171	0.000
CNN-DAE [36]	0.142±0.073	0.000	0.158±0.078	0.000	0.185±0.100	0.000	0.218±0.133	0.000
DeepFilter [28]	0.103±0.051	0.000	0.137±0.070	0.000	0.178±0.107	0.000	0.220±0.147	0.000
FCN-DAE [33]	0.108±0.059	0.000	0.130±0.070	0.000	0.167±0.103	0.000	0.215±0.152	0.000
ACDAE [34]	0.084±0.048	0.000	0.113±0.061	0.000	0.150±0.092	0.000	0.195±0.138	0.000
CBAM-DAE [36]	0.085±0.047	0.000	0.118±0.066	0.000	0.163±0.107	0.000	0.215±0.155	0.000
TCDAE [38]	0.085±0.051	0.000	0.115±0.066	0.000	0.157±0.104	0.000	0.207±0.150	0.000
FGDAE, q = 1	0.079±0.047	–	0.105±0.058	–	0.142±0.092	0.000	0.189±0.142	0.000
FGDAE, q = 2	0.079±0.048	0.000	0.105±0.059	0.919	0.139±0.088	–	0.183±0.133	–
FGDAE, q = 3	0.086±0.048	0.000	0.116±0.061	0.000	0.154±0.094	0.000	0.205±0.140	0.000
FGDAE, q = 4	0.121±0.069	0.000	0.166±0.083	0.000	0.216±0.115	0.000	0.261±0.138	0.000

* The bold black values indicate the best performance for each noise amplitude range.

**Table 11:** MAE in different noise segments for different methods for RMN experiment.

Method *	0.2to0.6	*p*-Value	0.6to1.0	*p*-Value	1.0to1.5	*p*-Value	1.5to2.0	*p*-Value
FIR Filter [28]	0.089±0.048	0.000	0.119±0.057	0.000	0.150±0.076	0.000	0.177±0.090	0.000
IIR Filter [28]	0.089±0.048	0.000	0.119±0.057	0.000	0.150±0.076	0.000	0.177±0.090	0.000
DRNN [27]	0.087±0.042	0.000	0.112±0.053	0.000	0.140±0.077	0.000	0.169±0.103	0.000
CNN-DAE [36]	0.091±0.048	0.000	0.102±0.051	0.000	0.120±0.066	0.000	0.143±0.090	0.000
DeepFilter [28]	0.078±0.038	0.000	0.100±0.049	0.000	0.124±0.068	0.000	0.149±0.090	0.000
FCN-DAE [33]	0.077±0.043	0.000	0.091±0.048	0.000	0.112±0.067	0.000	0.142±0.097	0.000
ACDAE [34]	0.062±0.035	0.000	0.080±0.043	0.000	0.102±0.060	0.000	0.129±0.089	0.000
CBAM-DAE [36]	0.064±0.035	0.000	0.086±0.047	0.000	0.114±0.072	0.000	0.147±0.104	0.000
TCDAE [38]	0.063±0.037	0.000	0.082±0.047	0.000	0.108±0.070	0.000	0.139±0.101	0.000
FGDAE, q = 1	0.059±0.035	–	0.076±0.042	–	0.099±0.062	0.000	0.128±0.093	0.000
FGDAE, q = 2	0.059±0.036	0.000	0.076±0.043	0.203	0.097±0.059	–	0.124±0.088	–
FGDAE, q = 3	0.064±0.036	0.000	0.084±0.043	0.000	0.108±0.063	0.000	0.139±0.095	0.000
FGDAE, q = 4	0.089±0.048	0.000	0.119±0.057	0.000	0.150±0.076	0.000	0.177±0.090	0.000

* The bold black values indicate the best performance for each noise amplitude range.

**Table 12:** SNR in different noise segments for different methods for RMN experiment.

Method *	0.2to0.6	*p*-Value	0.6to1.0	*p*-Value	1.0to1.5	*p*-Value	1.5to2.0	*p*-Value
FIR Filter [28]	7.070±2.458	0.000	4.212±2.313	0.000	2.040±2.512	0.000	0.333±2.688	0.000
IIR Filter [28]	7.070±2.458	0.000	4.212±2.313	0.000	2.040±2.512	0.000	0.333±2.688	0.000
DRNN [27]	6.753±2.600	0.000	4.305±2.597	0.000	2.316±3.035	0.000	0.534±3.393	0.000
CNN-DAE [36]	5.457±1.705	0.000	4.570±1.916	0.000	3.384±2.452	0.000	2.108±3.153	0.000
DeepFilter [28]	8.144±2.603	0.000	5.887±2.948	0.000	3.951±3.457	0.000	2.269±3.901	0.000
FCN-DAE [33]	7.873±2.496	0.000	6.307±2.795	0.000	4.503±3.440	0.000	2.566±4.089	0.000
ACDAE [34]	10.060±2.803	0.000	7.597±3.228	0.000	5.437±3.798	0.000	3.362±4.308	0.000
CBAM-DAE [36]	9.991±2.769	0.000	7.251±3.314	0.000	4.890±4.058	0.000	2.699±4.618	0.000
TCDAE [38]	10.015±3.193	0.000	7.541±3.540	0.000	5.191±4.177	0.000	2.998±4.664	0.000
FGDAE, q = 1	10.722±2.918	–	8.224±3.176	0.140	6.003±3.835	0.000	3.760±4.483	0.000
FGDAE, q = 2	10.657±2.921	0.002	8.256±3.187	–	6.097±3.680	–	3.922±4.243	–
FGDAE, q = 3	9.880±2.645	0.000	7.273±2.807	0.000	5.094±3.358	0.000	2.827±3.902	0.000
FGDAE, q = 4	7.070±2.458	0.000	4.212±2.313	0.000	2.040±2.512	0.000	0.333±2.688	0.000

* The bold black values indicate the best performance for each noise amplitude range.

**Table 13:** Error metrics for different noise combinations for TCDAE method [38].

Noise Type	SSD	MAD	PRD	Cosine Sim	RMSE	MAE	SNR
Clean	2.685±4.615	0.289±0.216	26.62±15.695	0.979±0.029	0.059±0.042	0.041±0.029	13.768±3.508
BW	7.442±12.116	0.429±0.335	44.69±25.698	0.909±0.100	0.102±0.065	0.074±0.045	8.561±3.777
MA	9.109±14.581	0.558±0.441	45.56±24.783	0.901±0.103	0.112±0.072	0.077±0.047	7.918±3.758
BW+MA	8.061±12.012	0.508±0.382	44.70±22.799	0.907±0.094	0.107±0.065	0.075±0.044	8.147±3.657
EM	25.10±49.390	0.718±0.566	66.70±35.187	0.768±0.229	0.177±0.134	0.122±0.090	4.561±4.845
BW+EM	24.46±45.362	0.713±0.566	65.89±36.233	0.770±0.234	0.175±0.131	0.120±0.088	4.739±5.048
MA+EM	23.38±45.079	0.720±0.566	63.84±32.877	0.783±0.215	0.170±0.129	0.117±0.087	4.759±4.804
BW+MA+EM	22.74±44.792	0.709±0.559	62.98±33.595	0.788±0.215	0.168±0.127	0.115±0.085	4.978±4.900

**Table 14:** Error metrics for different noise combinations for method (FGDAE, q = 1).

Noise Type	SSD	MAD	PRD	Cosine Sim	RMSE	MAE	SNR
Clean	2.101±4.448	0.183±0.161	20.96±14.373	0.981±0.024	0.050±0.040	0.038±0.029	15.293±3.817
BW	6.514±11.635	0.387±0.319	43.20±23.989	0.916±0.093	0.095±0.060	0.070±0.041	9.087±3.479
MA	7.914±13.924	0.491±0.399	46.52±26.585	0.905±0.099	0.104±0.068	0.072±0.046	8.559±3.455
BW+MA	7.006±11.417	0.447±0.343	45.21±24.200	0.911±0.089	0.100±0.061	0.071±0.043	8.768±3.421
EM	20.37±45.003	0.632±0.524	62.19±31.799	0.797±0.205	0.159±0.120	0.111±0.080	5.375±4.587
BW+EM	20.70±43.394	0.635±0.552	61.10±33.638	0.798±0.214	0.159±0.123	0.110±0.081	5.584±4.918
MA+EM	19.58±46.197	0.633±0.532	60.58±30.613	0.809±0.195	0.153±0.121	0.106±0.081	5.660±4.635
BW+MA+EM	18.85±41.868	0.629±0.531	60.28±31.441	0.810±0.199	0.152±0.117	0.105±0.076	5.844±4.719

**Table 15:** Error metrics for different noise combinations for method (FGDAE, q = 2).

Noise Type	SSD	MAD	PRD	Cosine Sim	RMSE	MAE	SNR
Clean	2.797±5.338	0.214±0.163	25.59±15.486	0.973±0.029	0.061±0.042	0.046±0.032	13.402±3.306
BW	6.760±11.892	0.397±0.338	43.35±28.492	0.917±0.092	0.097±0.062	0.070±0.042	9.033±3.501
MA	8.238±14.337	0.497±0.398	46.23±25.598	0.902±0.104	0.106±0.069	0.075±0.047	8.367±3.533
BW+MA	7.363±11.416	0.456±0.343	45.02±24.041	0.908±0.092	0.102±0.063	0.073±0.044	8.573±3.489
EM	18.484±41.76	0.612±0.502	62.06±33.132	0.804±0.202	0.153±0.113	0.107±0.075	5.680±4.451
BW+EM	18.186±38.61	0.604±0.508	61.62±35.891	0.806±0.207	0.151±0.112	0.106±0.075	5.842±4.643
MA+EM	18.250±43.05	0.617±0.517	60.73±32.032	0.812±0.193	0.149±0.116	0.104±0.077	5.792±4.471
BW+MA+EM	17.815±40.80	0.613±0.518	60.12±32.547	0.815±0.196	0.148±0.113	0.103±0.075	6.009±4.590

**Table 16:** Total number of parameters and testing times for selected models.

Method	Parameters	Testing Time (nv1)	Testing Time (nv2)
FCN-DAE	80.77 k	2.790 s	3.098 s
ACDAE	94.87 k	4.092 s	4.364 s
CBAM-DAE	217.51 k	7.417 s	7.621 s
TCDAE	1002.43 k	9.497 s	10.437 s
FGDAE, q = 1	263.25 k	9.560 s	10.900 s
FGDAE, q = 2	384.50 k	10.555 s	8.705 s

## Data Availability

The databases presented in this study (QT Database (QTDB) and MIT-BIH Stress Test Database (NSTDB)) are publicly available in Physionet at https://www.physionet.org/content/qtdb/1.0.0/ (reference number 10.13026/C24K53, accessed on 26 January 2025) and https://www.physionet.org/content/nstdb/1.0.0/ (reference number 10.13026/C2HS3T, accessed on 26 January 2025), respectively.

## Abbreviations

The following abbreviations are used in this manuscript:

CVDs	cardiovascular diseases
ECG	electrocardiogram
BW	baseline wander
MA	muscle artifact
EM	electrode motion
CNN	convolutional neural network
self-ONN	self-organized operational neural network
DAE	denoising autoencoder
ECA	efficient channel attention
CBAM	convolutional block attention module
GCN	gated convolutional network
nv1	noise split version 1
nv2	noise split version 2
RMN	random mixed noise
SSD	sum of squared distance
MAD	maximum absolute distance
PRD	percentage root-mean-square difference
CosSim	cosine similarity
RMSE	root mean square error
MAE	mean absolute error
SNR	signal-to-noise ratio
